# Experiences of frontline Pakistani emigrant physicians combating COVID-19 in the United Kingdom: a qualitative phenomenological analysis

**DOI:** 10.1186/s12913-021-06308-4

**Published:** 2021-03-31

**Authors:** Javeria Saleem, Muhammad Ishaq, Rubeena Zakar, Imran Hussain Khan Suddahazai, Florian Fischer

**Affiliations:** 1 Department of Public Health, Institute of Social and Cultural Studies, University of the Punjab, Lahore, Pakistan; 2 Department of Sociology, Institute of Social and Cultural Studies, University of the Punjab, Lahore, Pakistan; 3 Markfield Institute of Higher Education, Leicester, UK; 4 Institute of Public Health, Charité – Universitätsmedizin Berlin, Berlin, Germany; 5 Institute of Gerontological Health Services and Nursing Research, Ravensburg-Weingarten University of Applied Sciences, Weingarten, Germany

**Keywords:** Coronavirus, COVID-19, Pandemic, Emigration, Medical doctor

## Abstract

**Background:**

This study aims to explore the experiences, beliefs, feelings, and challenges faced by Pakistani migrant doctors working in the United Kingdom in times of the COVID-19 pandemic. The qualitative study aims to explore the lived experiences, beliefs, feelings, and challenges faced by Pakistani migrant physicians working in the United Kingdom during the COVID-19 pandemic.

**Methods:**

An exploratory phenomenological approach was used to collate data on experiences expressed during the COVID-19 pandemic. Purposive and snowball sampling was used to target participants, which were doctors of Pakistani origin involved in the direct care and management of COVID-19 patients in different NHS hospitals of the United Kingdom. Semi-structured, in-depth telephonic interviews were conducted with study participants in May 2020. Data analysis was done parallel with data collection by using an inductive qualitative approach.

**Results:**

We recruited ten frontline physicians. Four theme categories emerged from the data analysis: 1) Working across borders and cultures, 2) Role of beliefs for coping with stress and fear, 3) Passion and profession, and 4) Scaffolding the Pakistani health system. Overall, the results show that the participants received limited professional support, in terms of counseling and psychological rehabilitation. Instead, they had to use self-management strategies to cope with the situation.

**Conclusion:**

The intensive work exhausted participants physically and emotionally. They were holding a lot of grief and hurt inside, but still, healthcare professionals showed the spirit of professional dedication to overcome difficulties. Although currently coping with their emotional problems, comprehensive professional support should be made available to cater to the wellbeing of frontline physicians.

**Supplementary Information:**

The online version contains supplementary material available at 10.1186/s12913-021-06308-4.

## Background

The Coronavirus disease 2019 (COVID-19), a resultant of the severe acute respiratory syndrome coronavirus 2 (SARS-CoV-2), is an infectious disease that was first identified in the Chinese city of Wuhan in 2019 [1, 2]. The initial outbreak of COVID-19 became a rapidly evolving situation, as the disease escalated from a local epidemic in Wuhan to a global pandemic in a relatively short period. The World Health Organization (WHO) declared COVID-19 to be a public health emergency of international concern in January 2020 and then in March 2020 as a pandemic [3, 4]. At the end of August 2020, the number of death passed 820,000 people, out of a total of 25 million cumulative confirmed cases directly attributable to COVID-19 globally. The United Kingdom (UK) reported more than 330,000 cases and more than 40,000 deaths [5].

Due to the nature and speed of the pandemic, an immediate and intentional focus has been placed upon the core medical resources of each nation. The role of healthcare providers as valuable assets in both emergency and mundane situations has to lead to intense scrutiny and research upon the physical and psychological aspects of their health and security [6]. Initial research is already detailing the prevailing stress and unprecedented working conditions placed upon physicians and nursing staff, due to the highly contagious nature of the disease, its virulence, workload, uncertainty, stigma, and fear of spreading the infections to their family and loved ones [7].

Furthermore, previous studies have demonstrated that frontline physicians managing patients with COVID-19 have profound risks of suffering from mental ailments and disorders such as stress, insomnia, depression, and anxiety [8]. Research shows that these issues must be addressed immediately by providing healthcare workers with physical and psychological support during and post-crisis; especially with regards to the foreign physicians operating away from their regular family support, network, and familiarity these disorders have the propensity to intensify [9, 10].

Medical migration is a complicated and multidimensional global phenomenon, which is closely entwined with medical education. Medical migrants comprise a substantial ratio of the medical staff in numerous developed countries, referred to as ‘recipient countries’ [11]. The National Health Service (NHS) of the United Kingdom (UK) is the centralized healthcare provider in the UK and provides training and employment for a significant number of foreign doctors. The NHS workforce consists of around 12% foreign workers, of which 6% were of European citizenry and 6% non-European. South Asian doctors are by far the largest overall percentage of foreign doctors in the service [12].

Specifically, a large proportion of physicians from Pakistan (around 13,000) have migrated to the UK with the ambitions of completing their post-graduation qualifications, improving their economic status, and gaining employment and access to better facilities and resources [13]. These doctors of Pakistani origin are now working as frontline workers for the NHS to counter the COVID-19 outbreak in the UK. As a result of their work, some doctors have been affected due to the proximity and direct contact with patients of COVID-19, which has resulted in increased transmission within hospitals, self-isolation of doctors with little or no support, and tragically the deaths of several Pakistani doctors due to COVID-19 itself [14, 15].

However, despite the danger and hardship, Pakistani physicians have been able to help in combatting COVID-19 in Pakistan by collaborating with their colleagues through sharing their experiences and providing guidance on best practices and methods in treating COVID-19 patients. Moreover, the UK’s COVID-19 peak was earlier than Pakistan and these physicians were more acquainted to give valuable suggestions to their associates in Pakistan [16].

Delivering health services during this pandemic poses great threats to human relations, behaviour, emotions, and mental states that cannot be solely qualified or analysed through the exclusive deployment of quantitative studies. In order to uncover and capture the rich, meaningful experience of the practitioners, the discussion requires a qualitative approach [17]. A review of the literature revealed that there exists no qualitative research on the experiences of migrant Pakistani healthcare workers within the NHS during this COVID-19 pandemic. Therefore, this qualitative study aims to explore the lived experiences, beliefs, feelings, and challenges faced by Pakistani migrant physicians working in the UK during the COVID-19 pandemic.

## Methods

### Study design

An exploratory phenomenological approach was used to collate the experiential data of frontline Pakistani physicians working with COVID-19 patients in the UK. This approach was chosen in the data collection procedure to narratively explore the lived experiences of physicians of Pakistani origin involved in the direct care of COVID-19 patients. The phenomenological approach facilitates the researcher to explore Pakistani physicians’ views and concepts in combating the COVID-19 pandemic.

### Sampling and participants

Ten clinical frontline physicians of both genders working in the COVID-19 wards in different hospitals across the UK were selected to participate in the study. To attain diversity in experiences of study participants, considerations were taken with regards to variation in working experience, gender, age, duration of their total stay in the UK, family support, and place of employment.

A two-type sampling technique was used for data collection. In the first stage, a purposive sampling technique was used to collect the data from the participants following the snowball sampling technique. Three study participants were selected using purposive sampling, due to personal acquaintance, whilst the other seven were selected via snowball sampling.

### Interview guide

Semi-structured, in-depth telephonic interviews, of about 30 to 45 min’ duration each, were conducted with participants in the last week of May to first week of June 2020 by the second author (who has a Phd in Sociology with vast experience of conducting qualitative interviews) while he was on a short trip to the UK. Each interview was audio-recorded alongside the documentation of participant’s socio-demographic data. A semi-structured in-depth interview guide (see Additional file 1) was used for data collection. The guide was developed on the basis of extensive review of relevant literature and informal discussions with the physicians working in the COVID-19 wards in the UK hospitals. Furthermore, the interview guide was pilot tested to make sure that the questions were correctly worded to extract the relevant information.

### Data analysis

Data analysis was done in a convergent parallel design to data collection. The audio recordings were transcribed verbatim on the same day by authors and reappraised with the interviewees for accuracy. All the highlighted quotations and attributions of comments were approved by the participants.

**Fig. 1:**
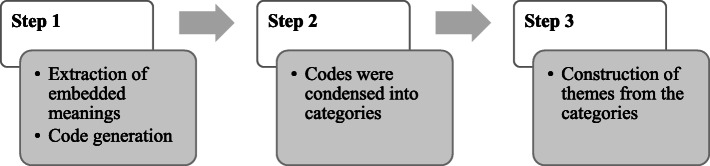
Flowchart describing the data analysis procedure

All data was tape-recorded in the native language of the participants. The voice recordings were transcribed by authors and then translated into the English language by authors. The transcribed data were read and reviewed multiple times individually by the study authors. The data were characterized into different codes (step 1) to extract the embedded meanings in the sentences. The second step (step 2) was selective coding, to finalize the important codes and transforming them into categories. Finally, thematic coding finds the relationship between codes to categorize and develop the themes (step 3) as shown in Fig. 1. These findings were peer-reviewed by the first four authors. The study authors met to discuss the identifies themes.

Trustworthiness and procedural consistency were formed to ensure the validity and reliability of the study findings [18] by ‘*Prolonged engagement’* and ‘*Persistent observation’* and tape recording the interview. Credibility was attained by checking interview statements in the data collection process. Transferability was determined by a full depiction of the research context and analytical generalization.

### Ethical considerations

The study was approved by the institutional review board of the University of the Punjab. The research has also been conducted in accordance with the recommendations of The British Educational Research Association guidelines. All participants were fully briefed and informed about the objective of the research. Both written and oral consent was attained from the participants before data collection. Pseudonymity of all participants was maintained through the use of an alphanumeric coding system (i.e. P1, P2) and the deleting of all identifiable data from the transcripts.

## Results

From the overall ten study participants, six were male and four female doctors. Four self-emergent themes were identified from the interviews and the subsequent data analysis: 1) Working across borders and cultures: This theme became apparent in every interview as participants continuously focused upon cultural and other issues faced by migrant physicians during the pandemic; 2) Role of belief as a coping strategy for stress and fear: This theme demonstrated the power of faith and belief in handling stressful and high pressured environments; 3) The application of passion and professionalism amidst dealing with critical COVID-19 patients: This theme emerged as a result of the participants increased awareness and appreciation of the standards and expectations of physicians in the UK; 4) Scaffolding the Pakistani health system: This theme emerged from the data due to the participants’ desires to translate, replicate and transfer the processes they had experienced with the NHS in their home country of Pakistan, specifically in dealing with COVID-19 cases.

### Working across borders and cultures

Working across borders and cultures is a multidisciplinary theme that explains the healthcare practices from a socio-public health perspective and cross-cultural approaches. P1 is a female doctor working in a hospital in the north of England. She has been residing and working in the UK for less than a year. Her previous professional experience and education have all been limited to Pakistan. When asked to reflect upon her experience in the UK, she replied with a deep sigh: *“Ah, there is a huge difference in everything, from personal to professional, my life got upside down, I was only hijabi doctor in the ward, my English language accent was different had issues both to understand and to convey my message, I almost lost my confidence”.*

She then proceeds to explicate upon some of the challenges she has identified in transitioning to life in the UK: *“Life in the UK is not as easy as I used to think in Pakistan …*”.

When probed further, she elaborates that “*We had a maid at home and a driver to pick kids from the school, but here one has to do everything by herself …”* .

These sentiments were shared by P2, another relatively new female doctor to the UK and the NHS, as she also complained about the workload and high levels of stress both in private and public life as compared to Pakistan.

The overarching perspectives of the doctors demonstrated that their experience of working in Pakistan and UK hospitals was completely different. P6 almost apologetically shares: *“I am sorry, but when I started working here a few years back, it seemed like I had learned nothing before, even I had done house job and a year service in Pakistan …” .*

A male doctor (P8) in a similar tone observed that the *UK health system is more structured and as a doctor, you have many liabilities, when new doctors come across this they get stressed especially when they are already struggling to settle down …”* .

The more experienced doctors presented a more in-depth perspective and recognized the diverse nuances that exist in UK society. P5, an experienced male doctor, noted that *“Pakistani and UK cultures are different but it’s not about the UK culture, there are many other cultures and ethnic groups we have to learn and work with”.*

P4, with extensive experience of working in Saudi Arabia, echoes this point and advises that *“if one is always ready to learn and adopt new things in life he can live and work easily in the UK”*.

In general, the doctors recognized the difference in the culture, living, and professional practices from Pakistan to the UK. Although some struggled to reconcile the new challenges they encountered, the experienced professionals appreciated the resourceful, systematic, professional environments they worked in as opposed to what they had experienced in Pakistan.

Overall, all the doctors commended the valuable institutional value of the NHS and what a positive impact it had made on their careers.

### Role of beliefs for coping with stress and fear

This theme explored the coping strategies used to spend conscious efforts and energy to solve personal and interpersonal problems in dealing with the COVID-19 pandemic. The overriding perspective appeared to demonstrate that the Pakistani doctors believed that their belief and reliance on their faith system and Allah, in particular, was central in helping them to cope with the situation. A female doctor (P7), for instance, stated that “*I call my family every day and this helps me a lot, another thing is turning to Allah, I do my best and then leave to Allah to protect me”.*

P1 shares that she was so fearful that she applied for the annual leave, her parents insisted that she should not work in the current situation “*but then a turning point came. I prayed to Allah and decided to jump into the situation and fulfill my oath, a soldier should never run out of the battlefield”.*

Furthermore, P3 notes that she was sure she would come across the virus while treating the patients, but “*I reminded myself that only what Allah wants will happen, as I don’t have an extended family system here to share my fears, my connection to Allah became stronger during these times”.*

Every participant emphasized the role of their beliefs to overcome fear and anxiety. P2 observes that *“in the beginning, I was numb. I knew I will carry the virus home too. I was also worried about my husband and child, I was reading Surahs [chapters] from the Quran to protect me …”.*

This reliance on Allah and their faith are further evident in P3’s experience: *“I was crying on the daily basis, patients were slipping from my hands [dying], the only console I had was to turning to God and seek help for my inner peace.”*

This demonstrates the fear caused by the COVID-19 pandemic. Physicians were describing their supposed everyday duties in terms of being akin to frontline soldiers. They were resorting to the sole reliance on God thus indicating a loss of trust and hope in the natural mundane physical world and a preparation for the one to come.

### Passion and profession

This theme explains the driving force which keeps the physicians passionate to work professionally in the COVID-19 wards. The participants were keen to discuss the set standards and procedures being deployed in the COVID-19 wards to treat patients. Overall, the doctors observed that the UK health system had failed to meet the needs of the physicians and patients because many of them had to work without even basic protective equipment, P2 shares: *“When this pandemic started, we took preventive measures like we have to sanitize everything before coming back to home, avoid contact with our family, stay at distance.”*

P3 also had similar feeling*s: “Despite all the PPEs, the fear of being affected and the risk factor is constantly with us. You don’t feel safe there at all.”* P1 describes her new experience: *“I spend almost one hour to prepare and change put clothes in the proper bags, life is not that straight easy as it used to be.”* P4 adds: *“It took time to become used to these PPEs, sometimes we treat the victim of the coronavirus without any precautionary measure. Another reason for this action is that we don’t consider the PPEs adequate and effective.”*

P1 shares that *“Sometimes I have to go over the prescribed protocols to treat the patients. Today I was with a COVID positive old lady, she tried to stand up and nearly fell but grabbed me I had to grab her too to assist.”*

This is a clear demonstration of the resulting conflict between ambiguous protocols and natural human concerns. The physicians were unanimous in declaring the precedence of humanity over professional protocol and prescribed standards, as illustrated by P2: “*We were all mentally prepared to face this pandemic but, in the beginning; we didn’t have proper PPEs, I can tell you I was one of the doctors who treated corona patients without any PPE.”*

However, adhering to the prescribed codes of conduct does not always entail ordinary activities but also the intangible essence of choosing between philosophical and psycho-social ideals. P1 in describing her experience reflects that she was discouraged by her Pakistani friends to not risk her life for the people who are not from her country and religion: *“I said I took an oath to treat human being not specific to any culture or religion. Even my religion says if you save one life it’s like saving whole humanity, there is no discrimination about the race colour, or religion.”*

P4 furthers the point by revealing that he is a religious person and has kept his beard for years but *“I had to shave it to fit the mask. When I am ready to risk my life for others, the beard is not a big deal, and I believe it’s not against Islam in these circumstances.”*

P8 supports this by sharing that the *“First time in my life I realized the importance of my medical profession where I can risk my life to save others.”*

The physicians were also unanimous in noting that the government’s support and campaigns, such as the creation of slogans to show appreciation for NHS staff and healthcare workers, and the special arrangement and priority are given to the doctors and other health workers in stores alongside the almost nationalistic Thursday 8 pm ‘clap for the NHS’ led to a positive image of the NHS and healthcare professionals and so the doctors were made to feel like heroes in their communities. P4 describes his feelings in this regard: *“The mass round of applause, different military terminologies used for the health workers by the general public and media is something quite new, it helps to boost our confidence. It is an acknowledgment of our role we are playing at this difficult time.”*

### Scaffolding the Pakistani health system

This theme shared the international experience with the native country to support the healthcare system. All of the doctors without question reflected upon how they would now try and contribute towards helping the Pakistani healthcare system to handle the COVID-19 crisis. P1 notes: *“If I have a chance, I will go to Pakistan and share my experiences with Pakistani colleagues.”*

Discussions involved the importance of training and showing seriousness to the growing cases.

P8 adds that his experience can be translated to a steep learning curve, which he wants to see replicated or at least the experience of it for his colleagues in Pakistan: *“Everyone knows there is no medicine for the disease, the role of nurses is more important in this case if Pakistani nurses can be trained it will help Pakistan.”*

P10 furthers the point: *“We should interview nurses and take their experiences to Pakistan, the role of the nurses in the UK has been sometimes more crucial than the doctors but in Pakistan, we don’t give much credit to the nursing staff.”*

Many of the participants reported being part of the Association of Pakistani Physicians of Northern Europe (APPNE) and doctHERs program which is an educational and training joint health initiative by the Pakistan Government and Pakistani health professionals in the UK to train and acquaint healthcare professionals in Pakistan on the latest knowledge and share health expertise related to treatment and prevention of COVID-19 through the use of technology.

The participants also lamented upon the fact that they thought the Pakistani people do not consider the disease to be serious. P5 is rueful in stating that he believes that *“People in Pakistan still think it’s a conspiracy and there is no real such disease. If they don’t take it seriously and follow social distancing guidelines, soon there will be dead bodies in the streets …”.*

He also observes that Pakistan’s health system is too weak and fragile to handle a critical mass of cases and will inevitably collapse, even if it records half of the cases the UK has had thus far. P5 was very angry about the incumbent government’s handling of the situation: *“Pakistani government is building castles in the air.”*

His concern is centered upon the notion that if richer, more resourceful nations such as those in Europe and the USA have been overwhelmed with the COVID-19 cases, how will a nation like Pakistan cope?

## Discussion

The study utilised a qualitative approach to get in-depth insights into the experiences of Pakistani migrant doctors combating COVID-19 in the UK. The findings concurred with discussions across the globe with regards to the lived experiences of healthcare workers combatting COVID-19 [19, 20]. They stated that it was their professional oath to work in any circumstances, irrespective of differences in culture or faith. The unpredictability and uncertainty surrounding the COVID-19 pandemic has caused major distress and worry for study participants, with fears ranging from catching the infection to infecting their loved ones and not being able to contain the contagion.

This also extends to their concerns for their immediate families in Pakistan. However, the data shows that they consider it against their work ethics to leave their duty and positions. Therefore, they will continue to deliver and uphold their medical oaths to the profession by fulfilling their responsibilities and developing a unifying spirit with their colleagues in the UK and Pakistan to combat the pandemic.

When the participants felt more experienced in dealing with COVID-19 related cases, due to its earlier peak in the UK [5], they were sure that Pakistan was not adequately equipped – neither infrastructurally nor organizationally – to handle the pandemic. Their insights into the Pakistani healthcare system have to lead them to begin online video consultations and training programs for healthcare professionals in Pakistan. They are working simultaneously to contain the disease in the UK, whilst preparing Pakistani healthcare professionals for a possible outbreak and containment in the future, thus scaffolding the development of Pakistani healthcare professionals. This can be considered an inadvertent benefit of a tragic situation.

However, despite this unnerving attitude towards the insurmountable challenges they are faced with, the participants revealed that in the early stages of the pandemic there existed tremendous anxiety with regards to the equipment and measures in place to protect both the healthcare workers and the patients. This issue was accentuated by the fact that no vaccination, cures, or medications exists and treatment is primarily reliant upon supportive nursing aid, psychological assistance, and prevention of complications [21].

In the initial phase of the COVID-19 pandemic, infection among healthcare workers had become a problem due to the unknown nature of the disease. Although measures had been taken, as they would be against any contagion, there existed uncertainty about its modes of transmission and signs of infection. Therefore, the participants undertook personal preventive measures such as sanitization of surfaces and sterilization of utensils and cooking items. Even the personal protective equipment (PPE) provided with the appropriate guidelines for use caused undue physical and emotional distress, due to the intensity of the work being undertaken in PPE gear for prolonged periods [19].

Female health workers in comparison with male respondents were in more frequent talks with their families in Pakistan and demonstrated more apprehension towards their patients in this outbreak. This finding was consistent with previous research related to immediate psychological response in the general public during the early phase of COVID-19. Furthermore, extensive epidemiological findings indicate that females were at high risk of depression than males [17, 22].

However, despite the issues encountered by the participants, the physicians may have not received any professional support, in terms of counseling and psychological rehabilitation. Instead, the strategies employed by the participants had to turn towards their faith. Previous studies have already substantiated this strong positive connection between religion and mental health. It was concluded that psychiatry and religion were closely connected and religious beliefs support an individual to sustain one’s life in different domains [23–25].

Furthermore, as well as relying upon their religion, the study participants have received unimaginable moral support and encouragement from the public at such a large scale which is unprecedented in the recent past. The participants acknowledged this as a major signifier in the advance towards a cure for mental health. A society that cares and appreciates all its members will help people feel wanted and cared for and thus boost their immune systems to help them fight diseases naturally [26].

Empirical evidence suggests that appreciation and gratitude have an invigorating effect on the brain for both expresser and receiver. Positive psychology studies have proven that appreciation and gratitude have a strong association with greater happiness because these feelings enhance dopamine production within the brain [27]. Dopamine is the same neurotransmitter that is released in the brain in reaction to when something good happens to us, i.e., having any reward or gift resulting in healthier sleep habits, boosting metabolism, and decrease stress levels [28, 29]. This underlines the importance of recognition and rewards towards the physicians and caregivers during the COVID-19 pandemic.

Some recommendations are suggested based on the current findings. The role of emigrant physicians in NHS is vital to meet the healthcare demands of the country. It is important to explore their living experiences to facilitate them with professional and emotional support. Mental health provisions should be made available such as professional psychological counseling and crisis intervention programs to cater to the wellbeing of the healthcare workers.

### Limitations

A major limitation of the research was that all respondents were interviewed via telephone. It was hard to build rapport with the interviewee on telephonic conversation and non-verbal cues might be missed. The shared experiences relate to a phase when the COVID-19 pandemic was still present and posing a large burden on the healthcare system. For that reason, opinions and attitudes may have changed over time, which we were not able to cover within this study.

Another limitation was the small sample size of the study. The data collection process was stopped when reached at saturation point, that was, at interview 10. The other reasons for this small sample size were that nothing was clear regarding the COVID-19 pandemic situation at that time and healthcare workers were facing such a challenge for the first time in their life, which restrict our access to the healthcare workers considering the strict lockdown in the UK, so we stopped the interviews at saturation point. Furthermore, this study was limited to physicians only and sample size could be improved if we would also include other healthcare professionals.

## Conclusion

This study explored the experiences of frontline Pakistani migrant doctors during the COVID-19 pandemic in the UK. In managing COVID-19 patients, Pakistani migrant physicians presented themselves as being profoundly professional, committed, and willing to place their own lives on the line to save others, a heroic and noble indictment of their efforts. In previous outbreaks such as SARS and MERS, clinical first-line healthcare workers were diagnosed with chronically stressed, anxiety, and depression disorders.

## Supplementary Information

**Additional file 1.**

## Data Availability

Data is available from the corresponding author upon reasonable request.

## Abbreviations

COVID: Coronavirus disease
NHS: National Health Service
PPE: Personal protective equipment
SARS: Severe acute respiratory syndrome
UK: United Kingdom
WHO: World Health Organization
