# Supplementary material for: Experiences of frontline Pakistani emigrant physicians combating COVID-19 in the United Kingdom: a qualitative phenomenological analysis
Source: BMC Health Serv Res. 2021 Mar 31;21:291. doi: 10.1186/s12913-021-06308-4 (PMC8011048; doi:10.1186/s12913-021-06308-4)
Supplement: Supplementary file 1 — Additional file 1. [file 12913_2021_6308_MOESM1_ESM.pdf]

Experiences of Frontline Pakistani Emigrant Physicians Combating COVID-19 in the United Kingdom: A Qualitative Phenomenological Analysis

Reading the consent to the participant

Demographic details / Introductory questions

Age:

Location in the UK:

Time living in the UK:

Living with family or alone:

Questions under three main themes

**Themes**	**Questions**
Working across borders and cultures: Problems and perspectives	• How long have been in this profession? (*Story of joining)*<br>• Do you have experience of working in Pakistan? *(How long? / How was it?)*<br>• Any working environment difference in the UK compared to Pakistan? (*Descriptions)*<br>• Do you face any cultural issues in the UK? (*How? / Why or any reasons?*)<br>• Do you think about Pakistan at this difficult time? (*Is it normal? Miss anything such as environment?)*
Coping with fear and anxiety	• How have you been chosen to work in the COVID-19 ward? (*Selection criterion)*<br>• What were your feelings to work in the COVID-19 ward?<br>• Can you tell if anything changed in your life?<br>• How safe do you feel working in the COVID-19 ward?

	• Which methods did you use to prepare yourself for this? (*spiritual/psychological)*<br>• Do you talk to your family back home? (*How often? Type of topics*)
Passion and profession	• What is more important according to your opinion in such conditions: Passion or profession?<br>• Have you ever tried to go over the prescribed limits to help patients?<br>• Do you think that protocols adopted for the patients and doctors are reasonable?<br>• Are there any suggestions you want to give for the Pakistani health system?<br>• Is there anything else you want to add into this?
